# Social Media Use Among Members of the Assessment of Spondyloarthritis International Society: Results of a Web-Based Survey

**DOI:** 10.2196/39155

**Published:** 2023-01-10

**Authors:** Yu Heng Kwan, Jie Kie Phang, Ting Hui Woon, Jean W Liew, Maureen Dubreuil, Fabian Proft, Sofia Ramiro, Anna Molto, Victoria Navarro-Compán, Manouk de Hooge, Bhowmik Meghnathi, Nelly Ziade, Sizheng Steven Zhao, Maria Llop, Xenofon Baraliakos, Warren Fong

**Affiliations:** 1 Program in Health Services and Systems Research, Duke-National University of Singapore Medical School Singapore Singapore; 2 Department of Rheumatology and Immunology, Singapore General Hospital Singapore Singapore; 3 Department of Pharmacy, National University of Singapore Singapore Singapore; 4 Section of Rheumatology, Boston University School of Medicine Boston, MA United States; 5 Division of Gastroenterology, Infectious Diseases and Rheumatology, Campus Benjamin Franklin, Charité - Universitätsmedizin Berlin Berlin Germany; 6 Department of Rheumatology, Leiden University Medical Center Leiden Netherlands; 7 Rheumatology Department, Cochin Hospital, Assistance Publique-Hôpitaux de Paris Paris France; 8 Rheumatology Service, University Hospital La Paz-IdiPaz Madrid Spain; 9 Rheumatology, Ghent University Hospital Ghent Belgium; 10 Department of Rheumatology & Clinical Immunology, Marengo Care Institute of Medical Sciences Hospital Ahmedabad India; 11 Department of Rheumatology, Saint-Joseph University, Hotel-Dieu de France Hospital Beirut Lebanon; 12 Centre for Epidemiology Versus Arthritis, Division of Musculoskeletal and Dermatological Sciences, School of Biological Sciences, Faculty of Biology Medicine and Health, The University of Manchester, Manchester Academic Health Science Centre Manchester United Kingdom; 13 Rheumatology, Parc Taulí Hospital Universitari Sabadell Spain; 14 Rheumazentrum Ruhrgebiet Herne, Ruhr University Bochum Germany; 15 Duke-National University of Singapore Medical School Singapore Singapore; 16 National University of Singapore, Yong Loo Lin School of Medicine, National University of Singapore Singapore Singapore

**Keywords:** social media, spondyloarthritis, cross-sectional survey

## Abstract

**Background:**

The use of social media in health care may serve as a beneficial tool for education, information dissemination, telemedicine, research, networking, and communications. To better leverage the benefits of social media, it is imperative to understand the patterns of its use and potential barriers to its implementation in health care. A previous study in 2016 that investigated social media use among young clinical rheumatologists (≤45 years) and basic scientists showed that there was substantial social media use among them for social and professional reasons. However, there is a limited inquiry into social media use in different areas of rheumatology, such as spondyloarthritis.

**Objective:**

We aimed to explore the motivations, barriers, and patterns of social media use among an international group of experts in spondyloarthritis.

**Methods:**

We distributed a web-based survey via email from March 2021 to June 2021 to 198 members of the Assessment of Spondyloarthritis International Society. It contained 24 questions about demographic characteristics, patterns of current social media use, and perceptions of utility. Univariable and multivariable logistic regression analyses were performed to identify the characteristics associated with use trends.

**Results:**

The response rate was 78.8% (156/198). Of these, 93.6% (146/156) of participants used at least one social media platform. Apart from internet-based shopping and entertainment, the use of social media for clinical updates (odds ratio [OR] 6.25, 95% CI 2.43-16.03) and research updates (OR 3.45, 95% CI 1.35-8.78) were associated with higher social media consumption. Among the respondents, 66% (103/156) used social media in a work-related manner. The use of social media for new web-based resources (OR 6.55, 95% CI 2.01-21.37), interaction with international colleagues (OR 4.66, 95% CI 1.21-17.90), and establishing a web-based presence (OR 4.05, 95% CI 1.25-13.13) were associated with higher levels of consumption for work-related purposes. Time investment, confidentiality concerns, and security concerns were the top 3 challenges to a wider adoption of social media.

**Conclusions:**

Most respondents (103/156, 66%) use social media in a work-related manner. Professional development, establishing a web-based presence, and international collaboration were associated with higher use. Challenges to social media adoption should be addressed to maximize its benefits.

## Introduction

### Social Media in Medicine

Social media has been increasing in popularity because of the accessibility, low cost, and availability of information on various topics. The number of social media users worldwide is postulated to increase from 4.26 billion in 2021 to 5.85 billion in 2027 [1,2]. In the health care sector, it is useful for education, information dissemination, research, networking, and communication for health care professionals and patients [3,4]. In recent years, health agencies and organizations have begun to leverage social media to obtain information for surveillance, correct medical misinformation, motivate changes in health behavior, and advocate for health-related issues [5]. Examples of social media use in the field of rheumatology include the monthly journal club on Twitter (#RheumJC), YouTube videos on rheumatic diseases [6,7], Facebook live videos of conferences [8], and participant recruitment for research studies [9]. In axial spondyloarthritis (axSpA), studies have demonstrated that the distribution of disease screening tools to high-risk patients over social media is an effective approach for a prompt diagnosis, especially because there is a lack of awareness of this disease among patients and health care professionals alike [10]. More extensive adoption of social media for clinical and research purposes may enable health care professionals to harness maximal benefits from social media, such as improving professional networking and education, organizational promotion, patient care, patient education, and public health programs [11-13].

### Social Media in the Era of COVID-19

During the COVID-19 pandemic, social media has become more important for communication and research in view of social distancing measures, travel restrictions, and the need to obtain new information within a short period. Over 50% of the existing Facebook groups were created during the pandemic to cater to the information needs of the public, with posts mostly covering topics such as general information about vaccination, side effects of vaccination, lockdown measures, and symptoms of COVID-19—information that was new and unique to the pandemic [14]. Clinical trials conducted remotely using social media recruitment strategies had a high retention rate (90.7%) and were able to include participants with a more diverse racial, ethnic, and geographical representation [15]. The COVID-19 Global Rheumatology Alliance web-based registry was set up in March 2020 by an international group of rheumatologists, data scientists, and patient partners to study the impact of the pandemic on patients with rheumatic disease and to identify patients at risk of having poorer outcomes after COVID-19 infection [16]. In situations where time is of the essence, social media would be able to facilitate the speed of knowledge acquisition and dissemination.

### Social Media in Spondyloarthritis

Although many medical organizations and agencies have developed social media platforms, there has been limited inquiry regarding the use of social media in rheumatology. A previous study in 2016 that investigated social media use among young clinical rheumatologists (≤45 years) and basic scientists showed that there was substantial social media use for social and professional reasons [17]. However, there has been no study on social media use in different areas in rheumatology, such as spondyloarthritis, which would be important as trends of social media use might vary across different specialties based on their unique job responsibilities and characteristics [18]. In addition, the onset of spondyloarthritis usually occurs between 20 and 30 years of age, an age group with better digital literacy, and inclined to use electronic platforms to research on health-related information [19,20]. Different diseases have different impacts on the patients’ quality of life and their requirements for support, which might influence their social media consumption [5,21]. Compared with other rheumatic diseases with apparent joint swelling or degeneration, axSpA mainly manifests in the spine and is associated with fatigue and unpredictable disease flares, which might not be readily understood by others [22,23]. Hence, patients with axSpA might be more inclined to seek social support and information on the web [23,24]. Diagnostic delay for axSpA is also significantly longer compared with other types of inflammatory arthritis, possibly because of poor recognition of its disease presentation among primary care physicians and nonrheumatologists [25-28]. Experts in axSpA could disseminate information and raise awareness about this disease through social media to facilitate prompt diagnosis and treatment in patients, especially as targeted education of primary care physicians has been shown to improve the recognition of axSpA and referral rates by 50% and 70%, respectively [29]. Screening questionnaires for axSpA could also be made available on social media for patients who could be prompted to visit a rheumatologist if they have a suspected diagnosis [30].

### Negative Aspects of Social Media

Despite the benefits of social media, its use is accompanied by certain risks, such as confidentiality breaches, display of unprofessional conduct, damage to professional reputation, and legal implications, if users are not equipped with knowledge about the appropriate use of social media [11,31]. There might also be difficulty in maintaining patient-physician relationships, where boundaries might be blurred [32,33]. Research participants’ recruitment rates might improve with the use of social media, but selection bias might be introduced if researchers do not fully understand the target population’s social media use and systematic study methodologies are not in place [32].

### Rationale and Aim of Study

To better capitalize on the benefits of social media, it is imperative to understand the trends of its use and views regarding its implementation in health care. The aim of this study was to explore (1) patterns, (2) reasons, (3) barriers to social media use, and (4) perceptions of utility among an international group of experts in spondyloarthritis. This study was initiated by Young-Assessment of Spondyloarthritis International Society (Y-ASAS), a platform within the Assessment of Spondyloarthritis International Society (ASAS) for members aged ≤45 years.

## Methods

### Design

An open web-based survey using Google Forms was distributed via email to all 198 members of the ASAS worldwide, who were experts in the area of spondyloarthritis and mainly consisted of clinicians and researchers. The survey was designed based on the Checklist for Reporting Results of Internet E-Surveys (CHERRIES) criteria [34]. Participants provided implied informed consent before completing the web-based questionnaire. Data were collected anonymously from March 2021 to June 2021, and the data analysis began in July 2021. No reminders were sent to the nonrespondents.

### Survey Questionnaire

The questionnaire for the survey was designed by the Y-ASAS. It was adapted from other studies [17,35,36] and included 2 sections with 24 questions (Multimedia Appendix 1). The first section concentrated on demographic characteristics such as age, sex, primary occupational role (eg, academic clinician or researcher), and level of social media knowledge (response type: numerical rating scale, where 0 meant very poor knowledge and 10 meant very good knowledge). The second section included questions regarding the use of social media, such as the type of social media platform used, frequency, purpose, and barriers of use, and perceptions of utility. Social media platforms include apps for messaging (eg, WhatsApp), social networking (eg, Facebook), media sharing (eg, YouTube), blogs, and microblogs (eg, Twitter). We beta-tested the survey for clarity and comprehensiveness with 2 members of the intended target audience before implementation.

### Statistical Analysis

Continuous variables are reported as means and SD if normally distributed or as median and IQR if otherwise. Categorical variables are reported as numbers and percentages. Reasons associated with (1) the use of social media for any purpose and (2) work-related purposes were identified using univariable and multivariable logistic regression, with the level of social media use (high vs low) per week as the dependent variable for both purposes 1 and 2. Multivariable analyses were adjusted for age (categorized into <50 years and ≥50 years), sex, primary occupational role, and level of social media knowledge [37,38]. The analysis for the second purpose was restricted to respondents who reported engaging in social media activities for work-related purposes. The level of social media use was categorized into low and high, based on the hours used per week. Participants who were above the 75th percentile for time spent on social media (above 12 hours per week when used for any purpose and above 5 hours per week when used in a work-related manner) were defined as having high levels of use; participants who fell below the 75th percentile (12 hours or less per week when used for any purpose and 5 hours or less per week when used in a work-related manner) were defined as having low levels of use [39]. The results of these analyses are reported as odds ratio (OR) and 95% CI. All statistical analyses were conducted using STATA SE 15 (StataCorp LLC).

### Ethical Considerations

The SingHealth Centralized Institutional Review Board approved this study (reference number: 2020/2006).

## Results

### Overview

Of the 198 ASAS members, 156 responses were received (156/198, 78.8% response rate). Most respondents were male (107/156, 68.6%), aged between 40 and 49 years (59/156, 37.8%), and working as academic clinicians (107/156, 68.6%). Members of more than 40 countries responded to this survey, and the majority were from Europe (104/156, 66.7%). When asked to rate their level of social media knowledge on a numerical rating scale from 0 (very poor knowledge) to 10 (very good knowledge), the median (IQR) rating was 6 (4-8). The majority (146/156, 93.6%) of the survey respondents reported using social media, with the median (IQR) time spent on it being 6 (2-12) hours per week (Table 1). A total of 66% (103/156) of respondents used social media in a work-related manner, with a median (IQR) use of 2 (0.5-5) hours per week (Table 1).

**Table 1:** Characteristics of respondents (N=156).

Demographics		Values
Male, n (%)		107 (68.6)
**Age (years), n (%)**		
	30-39	20 (12.8)
	40-49	59 (37.8)
	50-59	41 (26.3)
	60-69	19 (12.2)
	≥70	17 (10.9)
**Geographic region, n (%)**		
	Africa	4 (2.6)
	Asia and pacific	22 (14.1)
	Europe	104 (66.7)
	North America	16 (10.3)
	South America	10 (6.4)
**Primary role, n (%)**		
	Academic clinician	107 (68.6)
	Academic researcher	49 (31.4)
Knowledge of social media^a,b^, median (IQR)		6 (4-8)
**Use social media, n (%)**		146 (93.6)
	Time spent per week (hours), median (IQR)	6 (2-12)
	Low level of use (12 hours or less per week), n (%)	110 (75.3)
	High level of use (more than 12 hours per week), n (%)	36 (24.7)
**Use social media for work, n (%)**		103 (66)
	Time spent per week (hours), median (IQR)	2 (0.5-5)
	Low level of use (5 hours or less per week), n (%)	74 (71.8)
	High level of use (more than 5 hours per week), n (%)	29 (28.2)

^a^One participant did not respond.

^b^Respondents were asked to rate their level of social media knowledge on a numerical rating scale from 0 (very poor knowledge) to 10 (very good knowledge).

### Reasons for Social Media Use

**Figure 1:**
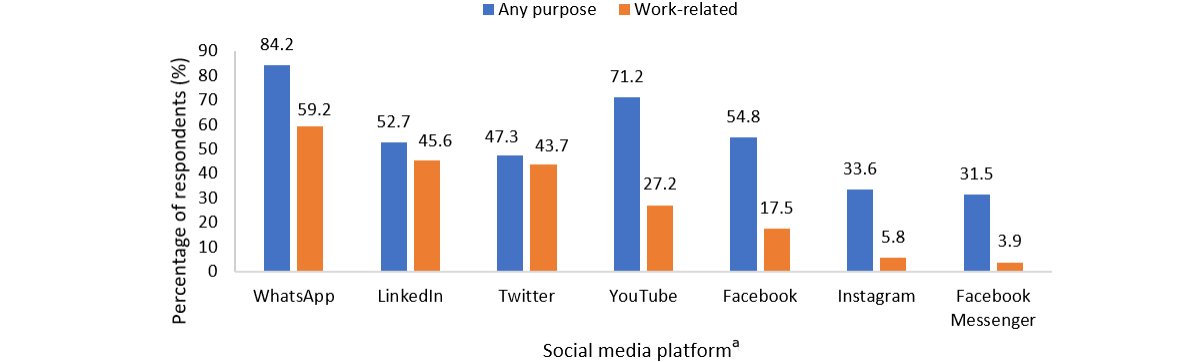
Use of social media platforms. ^a^Qzone, Sina Weibo, and Baidu Tieba were not included in the figure as none of the respondents used these platforms. Viber, Telegram, Snapchat, Pinterest, Tik Tok, Line, Reddit, Medium, QQ, and Tumblr were not included in the figure as less than 10% of respondents used these platforms.

Among the 146 respondents who used social media, the top 3 reasons were to catch up with friends (101/146, 69.2%), to network (81/146, 55.5%), and to socialize (75/146, 51.4%). The use of social media for clinical updates (OR 6.25, 95% CI 2.43-16.03), internet-based shopping (OR 5.95, 95% CI 1.59-22.25), research updates (OR 3.45, 95% CI 1.35-8.78), and entertainment (OR 2.72, 95% CI 1.06-6.97) were significantly associated with a higher level of social media activity, when adjusted for age, sex, primary occupational role, and level of social media knowledge (Table 2). WhatsApp (123/146, 84.2%), YouTube (104/146, 71.2%), and Facebook (80/146, 54.8%) were the top 3 social media platforms used (Figure 1).

**Figure 2:**
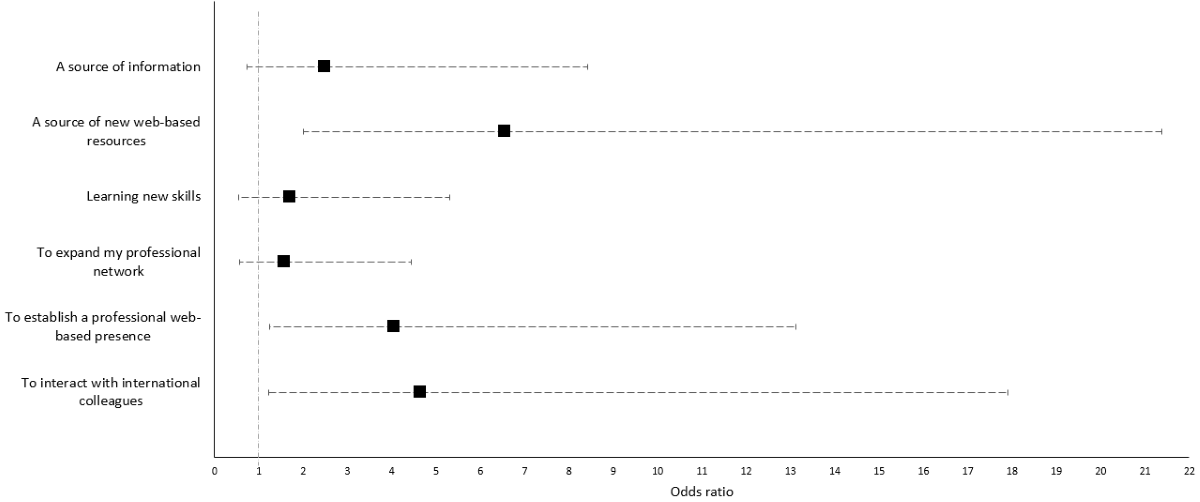
Associations between reasons for social media use in a work-related manner and level of social media use for work-related purposes, adjusted by age, sex, primary role and level of knowledge on social media.

Among the 103 respondents who used social media for work-related purposes, the top 3 reasons stated were to obtain information (69/103, 67%), interact with international colleagues (63/103, 61.2%), and expand their professional network (54/103, 52.4%). When using social media in a work-related manner, the use of social media for new web-based resources (OR 6.55, 95% CI 2.01-21.37), to interact with international colleagues (OR 4.66, 95% CI 1.21-17.90), and to establish a web-based presence (OR 4.05, 95% CI 1.25-13.13) were significantly associated with a higher level of social media use when adjusted for age, sex, primary role, and level of social media knowledge (Figure 2). WhatsApp (61/103, 59.2%), LinkedIn (47/103, 45.6%), and Twitter (45/103, 43.7%) were the top 3 social media platforms used for work-related purposes (Figure 1).

**Table 2:** Associations between reasons for social media use and level of social media use for any purpose.

			Level of social media use for any purpose		
			Univariable, OR^a^ (95% CI)		Multivariable^b^, OR (95% CI)
**Age (years)**					
	≥50	Reference		N/A^c^	
	<50	2.00 (0.91-4.39)		N/A	
**Sex**					
	Male	Reference		N/A	
	Female	0.99 (0.44-2.19)		N/A	
**Clinician or researcher**					
	Academic clinician	Reference		N/A	
	Academic researcher	0.64 (0.26-1.55)		N/A	
Level of social media knowledge			1.52 (1.22-1.90)		N/A
Catch up			1.37 (0.56-3.35)		0.93 (0.35-2.48)
Network			2.14 (0.94-4.91)		1.31 (0.52-3.27)
Socialize			2.70 (1.18-6.18)		1.87 (0.76-4.59)
Share knowledge			5.24 (2.10-13.09)		2.77 (0.98-7.79)
News update			2.61 (1.18-5.81)		1.67 (0.70-4.02)
Entertainment			3.22 (1.43-7.27)		2.72 (1.06-6.97)
Clinical update			8.80 (3.70-20.96)		6.25 (2.43-16.03)
Research update			5.92 (2.57-13.65)		3.45 (1.35-8.78)
Event update			3.33 (1.47-7.55)		2.01 (0.80-5.02)
Politics			3.00 (1.00-9.00)		1.93 (0.59-6.34)
Job update			4.10 (1.17-14.40)		3.59 (0.97-13.32)
Internet-based shopping			6.85 (2.12-22.20)		5.95 (1.59-22.25)

^a^OR: odds ratio.

^b^Multivariable analysis was adjusted for age, sex, primary role, and level of knowledge on social media.

^c^N/A: not applicable.

### Barriers to Use of Social Media

Among the 53 respondents who did not use social media for work-related purposes, the top 3 reasons cited were “Concerned about privacy” (24/53, 45%), “Not suitable for my needs” (23/53, 43%), and “Lack of knowledge on how to use social media” (20/53, 38%; Table 3).

**Table 3:** Barriers to use of social media in a work-related manner (n=53)^a^.

Barriers to use of social media in a work-related manner	Values, n (%)
Concerned about privacy	24 (45)
Not suitable for my needs	23 (43)
Lack of knowledge on how to use social media	20 (38)
Not interested	18 (34)
Concerned regarding the validity of information on social media	17 (32)
No time	16 (30)
Concerned regarding its safety	16 (30)
Concerned about negative impact on reputation	5 (9)
Others^b^	1 (2)

^a^Out of the respondents, 53 did not use social media in a work-related manner. One participant did not respond to question which asked about barriers to the use of social media.

^b^One participant mentioned that a potential barrier was because of the influence of artificial intelligence on the views.

### Perceptions of Utility of Social Media

**Figure 3:**
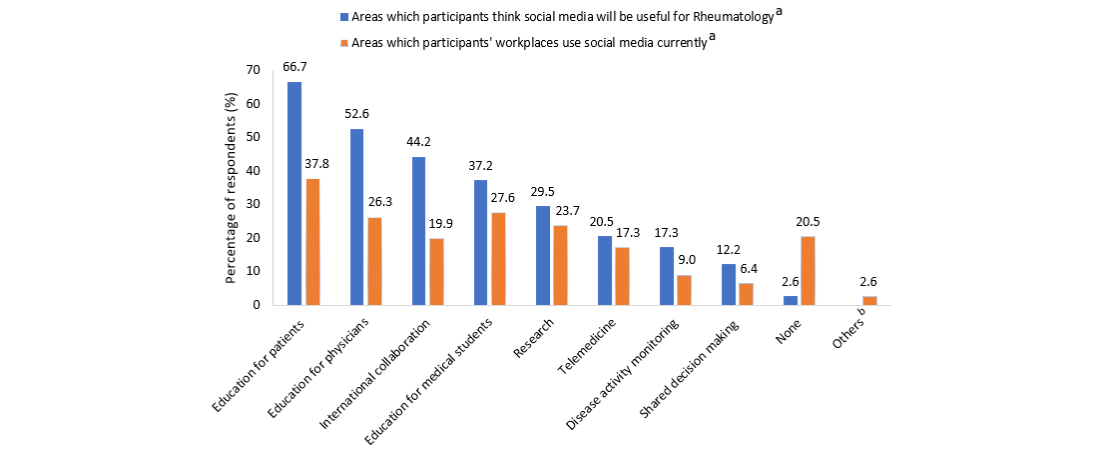
Perceptions of utility of social media. ^a^Out of the 156 participants, 8 participants did not respond to the question asking about areas in which social media will be useful for Rheumatology and 17 participants did not respond to the question asking about areas in which their workplaces use social media. ^b^One participant mentioned that it was used for official press releases, 3 participants mentioned that it was used for communication among staff.

The top 3 current uses of social media within the respondents’ workplaces were “Education for patients” (59/156, 37.8%), “Education for medical students” (43/156, 27.6%), and “Education for physicians” (41/156, 26.3%) (Figure 3). When asked about the potential uses of social media in rheumatology, the top 3 areas chosen were “Education for patients” (104/156, 66.7%), “Education for physicians” (82/156, 52.6%), and “International collaboration” (69/156, 44.2%; Figure 3). As for the recipients who would benefit the most if social media were used as a communication tool for rheumatology, the top 3 groups selected were patients (100/156, 64.1%), researchers (85/156, 54.5%), and health care providers in the community (80/156, 51.3%; Table 4). Social media was generally deemed less impactful in those aged >60 years.

When all respondents were asked about the challenges to a wider adoption of social media for rheumatology, the top 3 reasons were “Time investment” (91/156, 58.3%), “Confidentiality concern” (71/156, 45.5%), and “Security concern” (69/156, 44.2%; Table 5).

**Table 4:** Recipients whom respondents think would benefit from social media.^a^

Recipients who would benefit from social media	Values, n (%)
Patients	100 (64.1)
Researchers	85 (54.5)
Health care providers in the community	80 (51.3)
Caregivers	43 (27.6)
Health care providers in acute hospital	37 (23.7)
Others^b^	3 (1.9)

^a^Out of the 156 participants, 18 (11.5%) participants did not respond to the question.

^b^One participant mentioned that it was dependent on goal and medium used, and 2 participants mentioned that rheumatologists in a network would benefit.

**Table 5:** Challenges in the wider adoption of social media for rheumatology.^a^

Challenges in the wider adoption of social media for rheumatology	Values, n (%)
Time investment	91 (58.3)
Confidentiality concern	71 (45.5)
Security concern	69 (44.2)
Adoption by health care providers	52 (33.3)
Legal grounds	50 (32.1)
Infrastructure development of technological approaches	46 (29.5)
Supervision and follow-up	45 (28.8)
Adoption by patients	44 (28.2)
Information anarchy	40 (25.6)
Cost investment	30 (19.2)
Workplace acceptance and support	21 (13.5)
Inefficiency	21 (13.5)
Lack of skills	20 (12.8)
Others^b^	1 (0.6)

^a^Out of the 156 participants, 19 (12.2%) participants did not respond to the question.

^b^One participant mentioned health literacy.

## Discussion

To our knowledge, this is the first study to investigate the patterns, reasons, barriers, and perceptions of social media use among an international group of spondyloarthritis experts.

### Patterns of Social Media Use

Among the respondents, 93.6% (146/156) were users of social media, which falls within the range of 88% to 98%, as reported in previous studies [37,40,41]. The majority (103/156, 66%) of respondents used social media in a work-related manner. This value was similar to that reported by young rheumatologists and basic scientists [17] but was higher when compared with population health professionals [37]. Differences in the location and time at which the research study was conducted, as well as the nature of the respondents’ practices, could have resulted in the observed variations.

WhatsApp was the most commonly used platform for both general and work-related purposes, while Twitter and LinkedIn were the next most used platforms for work-related purposes. Prior studies have also concluded that WhatsApp is the most commonly used social media platform [18,37]. The majority of physicians communicate with their colleagues through WhatsApp, and 50% of them are part of professional WhatsApp groups, specifically for medical case discussions [42]. Owing to its low cost and convenience, WhatsApp is gaining popularity and has been shown to improve communication among health care professionals [43-45]. When health care professionals in obstetrics and gynecology were asked to rank their use of social media platforms (Twitter, Facebook, Hyves, YouTube, and LinkedIn), LinkedIn and Twitter were the top 2 social media platforms used for reasons such as networking and updating colleagues about their work [46]. Another survey showed that Twitter was the most frequently used platform for professional development and was viewed as the most useful for improving knowledge, problem-solving, creativity, and other domains related to professional development [47]. For Twitter, hashtags created for certain topics facilitate the sharing and discussion of information, even for rare diseases, and would be a good platform for networking and fostering collaboration [48,49]. Views on the popularity of LinkedIn, however, have shown more divergence in previous studies, possibly because of its branding as a platform for job search, which might have led users to neglect other potential uses (eg, education) more relevant to them [50].

### Reasons for Social Media Use

This study found that, besides entertainment and internet-based shopping, significant time was spent on social media platforms for clinical and research updates. The use of social media for new web-based resources (eg, YouTube videos, Twitter, and Instagram pages) to establish a professional web-based presence and to interact with international colleagues were associated with a higher level of use when used in a work-related manner.

On the basis of previous literature, these reasons are also commonly cited by health care professionals for social media use. Among Chinese urologists, the top 3 reasons for social media use were professional communication and collaboration, obtaining medical information, and communicating with patients; of these, its use for information acquisition increased the most (tripled) from 2014 to 2016 [51]. A survey of physicians, pharmacists, and allied health professionals revealed that over 50% of them used social media to exchange information and communicate with their colleagues [18]. Among physicians who use social media regularly, the main reasons for its use include communication with colleagues, personal branding, networking with local and international peers who share similar interests to promote professional collaborations, and sharing and obtaining updated knowledge [4]. These participants also used resources available on social media as a means of benchmarking to ensure that their skills (eg, practical procedures) were on par with other medical professionals and to learn from best practices [4].

Owing to the higher effectiveness of learning and speed of updates associated with social media use, it has been a popular choice for continued medical education compared with traditional methods of obtaining information (eg, journal papers) [4,52]. International collaborations also enhance the exchange of clinical information, as demonstrated through the International Collaborative Grand Rounds (ICGRx), where participants can interact and contribute to the discussions remotely [53]. Health care professionals also view web-based communities as a useful platform for obtaining information to help in making informed decisions [54]. When used in research, social media is a useful platform to encourage discussion and collaboration to allow research projects to be promptly set up [16]. Some researchers have also used social media as a strategy to boost participant recruitment, especially for recruitment targeted at minoritized or historically excluded groups or populations that are harder to reach [55,56] or if recruitment had to be carried out on a large-scale [57,58]. Health care professionals are also establishing a web-based presence on social media and using it as an advocacy tool to campaign for certain causes or to expel biases [59], or as a marketing tool to improve their competitiveness [60], especially as patients are turning to web-based sources for health-related information.

### Barriers to Use of Social Media

Among the respondents who did not use social media for work-related purposes, concerns about privacy, viewing social media as unsuitable for their needs, and lack of knowledge were among the top 3 barriers. On the basis of a systematic review, issues regarding privacy, data protection, and not being equipped with insufficient technical skills were also identified as deterrents against the use of networking sites [48]. Among surgical faculty and trainees, the top 3 reasons for the use of social media for professional purposes were a preference for more traditional methods of education, communication, collaboration, concerns about violations of personal privacy, and patient privacy [61]. An inclination toward more traditional methods of information acquisition and communication might have caused respondents to view social media as misaligned with their needs. Even among physicians who used social media to educate viewers about health information, a lack of knowledge on how to use social media was also a major barrier [62].

To tackle these issues, training regarding the appropriate use of social media could be implemented to inform medical professionals about the technicalities of various social media platforms, as well as ways to use social media to enhance learning, communication, and collaboration, without compromising privacy or their reputation (eg, adhering to guidelines on maintaining confidentiality, institutional policies, and adjusting privacy settings) [17,63,64]. Future studies could investigate the gaps in social media knowledge and evaluate different teaching interventions to develop more effective teaching plans. Late-career medical professionals could be encouraged to share their clinical knowledge and experience on social media, as they might be favored by younger physicians for knowledge acquisition [35,61]. When using social media, medical professionals should segregate education from personal posts to maintain a more professional image [65]. Physicians should also be mindful of protecting patients’ personal information and obtaining relevant consent before posting clinical data or photographs [66].

### Perceptions of Utility of Social Media

When asked about perceptions of the utility of social media, most of the respondents in our survey found it useful for patient education, which is broadly similar to a previous study where 61.5% of physicians encouraged their patients to search for information on their disease on the internet [41]. Information seeking was a primary objective for patients’ use of social media, and a survey showed that 95% of patients who visited an esthetic medicine clinic sought information on the internet about the procedures before their consultation, with social media being the first source for 46% of them [46,67]. Clinicians typically have limited consultation time with patients, and posting of health information written for a nonmedical audience on social media would allow patients to learn more about information relevant to their medical conditions, which might not be covered comprehensively during consultation [62]. This is especially relevant as patients currently play a more active role in decision-making and seek health information to be more informed [68,69]. Apart from information seeking, patients have also turned to social media for emotional and network support to better cope with the disease [24]. However, these conclusions are in contrast to a study conducted among population health stakeholders, who ranked patient adoption as the second main challenge against the use of social media, possibly because of differing patient profiles regarding age and motivations for use [37].

Respondents viewed time investment as the greatest challenge to the wider adoption of social media for rheumatology, while concerns about confidentiality and security were ranked second and third, respectively. Time constraints were also ranked as the top challenge faced by oncology physicians, physicians-in-training, and population health stakeholders regarding the use of social media [35,37]. On the basis of previous qualitative studies, contributing to social media was challenging because of the time required for preparation work (eg, research, referencing, editing of video, and posts), and physicians often managed their social media accounts at the expense of other commitments [4,62]. Furthermore, posting on social media to communicate health-related information would require medical professionals to spend more time crafting and checking the content to ensure that viewers do not misunderstand the information and prevent legal implications, a role that might not be compensated [62]. Concerns about a breach of their personal as well as patients’ privacy were also cited as primary reasons for being reluctant to dabble in social media in previous studies [70-72]. Despite these concerns, many health care workers are unaware of the content and availability of guidelines regarding the appropriate use of social media [41,63,73].

Compared with previous surveys on social media use among health care professionals [35,70], our study had a high response rate of 78.8%. Given the good response rate, wide age range, and international distribution of participants, the findings of this study can serve as a basis for future research and implementation. The diverse age range of the respondents is also a strength of this study, as it would facilitate the generalization of the results across various age groups. Although the data for this study were collected in the middle of the COVID-19 pandemic, which could have influenced the preferences and habits of social media use by the respondents, the study was designed in January 2020 before the pandemic. Hence, the areas of inquiry and results of this study could still be relevant after the pandemic.

This study had some limitations. There may be selection bias, as participants who responded to the email invitation and web-based survey may have been more inclined to use social media. However, the geographic distribution of respondents in this study is similar to that of ASAS members, suggesting that it would be a representative sample (Multimedia Appendix 2). Other demographic profiles of the ASAS members were also similar to those of the respondents in this study. Of all the ASAS members, 70% were male, similar to the 68.6% obtained in this study. The mean age of all ASAS members was 53.1 years, which fell within the age range of 50-59 years, the second most dominant age group of respondents in this study. This survey also only included clinicians and researchers specializing in spondyloarthritis and had a smaller sample size, with about two-thirds of the respondents practicing as academic clinicians, which may limit the generalizability of the study findings to professionals practicing in different specializations or clinical settings. In addition, ASAS members might assume different clinical roles and hence use social media differently, but we did not collect data regarding their job scope, which might restrict the analysis of the data. In addition, data were self-reported instead of being based on use statistics, and hence, prone to recall bias.

### Conclusions

In conclusion, this study conducted among clinicians and researchers with an interest in spondyloarthritis showed that the majority (66%) of respondents used social media for work-related purposes, such as international collaboration, establishing a professional web-based presence, and professional development. In addition, social media may be useful as an educational tool for both patients and physicians.

Efforts should be made to address the challenges to wider adoption of social media to encourage its use, given that patients with axSpA tend to be of a young age range (20-30 years) who might be more tech-savvy and inclined to search for health-related information on the internet. Despite this, rheumatology and health care organizations should also be aware of certain risks and disadvantages of implementing social media for professional use, such as the distribution of poor-quality information, breach of privacy (for patients and health care professionals), crumbling of patient-physician boundaries, and potential legal ramifications, if not used prudently. Training regarding the technical and ethical aspects of social media use can be conducted to give health care professionals more confidence in using social media. Guidelines for the appropriate use of social media should be developed, and their importance should also be highlighted. Professionals should also select an appropriate type of social media platform based on the aim of its use (eg, dissemination of information to medical professionals and research study recruitment).

## Appendix

Multimedia Appendix 1 Survey questions.

Multimedia Appendix 2 Geographical distribution of all Assessment of Spondyloarthritis International Society members.

## Abbreviations

ASAS: Assessment of Spondyloarthritis International Society
axSpA: axial spondyloarthritis
EMEUNET: Emerging Eular Network
MSD: Merck Sharp & Dohme
OR: odds ratio
UCB: Union Chimique Belge
Y-ASAS: Young-Assessment of Spondyloarthritis International Society
