# Supplementary material for: Social Media Use Among Members of the Assessment of Spondyloarthritis International Society: Results of a Web-Based Survey
Source: J Med Internet Res. 2023 Jan 10;25:e39155. doi: 10.2196/39155 (PMC9875001; doi:10.2196/39155)
Supplement: Multimedia Appendix 1 [file jmir_v25i1e39155_app1.docx]

Multimedia Appendix 1: Survey questions

*Background: In order to understand our members’ preferences for social media and newsletter content in order to tailor our efforts better, we would like to invite you to take about 7 minutes to participate in this short anonymous survey. Your participation is voluntary and you may stop at any time. Please be assured that no identifiable data would be collected in this survey.*

# Questionnaire

***Questions regarding social media use***

*Section A. We would like to ask about yourself and your experience to help us understand your responses better.*

**Please enter your type of ASAS membership:**

Associate ASAS member

Full ASAS member

Honorary ASAS member

**Would you consider yourself primarily a:**

Rheumatologist

Radiologist

Other health professional

**Are working primarily as an:**

Academic researcher

Academic clinician

**How long have you worked in the field of rheumatology (including clinical training)?** ______ year(s) [if less than a year, put ‘0’]

I do not work in rheumatology

**What is your sex?**

Male

Female

**What is your age?**

Below 30 years

30-39

40-49

50-59

60-69

70 years or over

**What is your country of residence?** _____________________

*Section B. The use of social media is growing rapidly, and we would like your thoughts on how it can be used in rheumatological practice. As members and contributors to the health system, your input is vital in helping us design initiatives that are relevant and impactful.* ***Social media*** *refers to websites and internet applications that allow users to create and share content or to participate in social networking.*

1. **How would you rate your knowledge of social media (e.g. what it includes, how to use it, its security level etc.)?**

| Very poor |  |  |  |  |  |  |  |  |  |  |  | Very good |
| --- | --- | --- | --- | --- | --- | --- | --- | --- | --- | --- | --- | --- |
|  | 0 | 1 | 2 | 3 | 4 | 5 | 6 | 7 | 8 | 9 | 10 |  |

**Comments:**

1. **I consider social media to be a safe way of communicating with other people.**

- Yes
- No

1. **Do you use social media?**

- Yes (Proceed to Q12)
- No

**If not, what is your reason for not using social media? (select all that apply)**

- Lack of knowledge on how to use social media
- Not suitable for my needs
- Concerned about privacy
- Concerned regarding its safety
- Concerned regarding the validity of the information on social media
- Concerned about negative impact on my reputation
- Not interested
- No time
- Others; please specify ___________________________________

(Proceed to Q18)

**Which social media platform do you use and how frequently do you use it? (select all that apply) Please state the number of hours of using the social media platform.**

|  | **Social Media Platform** | **Average hour per weekly use** | **Never** |
| --- | --- | --- | --- |
|  | Facebook |  |  |
|  | YouTube |  |  |
|  | Whatsapp |  |  |
|  | Facebook Messenger |  |  |
|  | Instagram |  |  |
|  | QQ |  |  |
|  | Tumblr |  |  |
|  | Qzone |  |  |
|  | Tik Tok |  |  |
|  | Sina Weibo |  |  |
|  | Twitter |  |  |
|  | Reddit |  |  |
|  | Baidu Tieba |  |  |
|  | LinkedIn |  |  |
|  | Viber |  |  |
|  | Snapchat |  |  |
|  | Pinterest |  |  |
|  | Line |  |  |
|  | Telegram |  |  |
|  | Medium |  |  |
|  | Others; please specify __________________ |  |  |

1. **Why do you use social media? (select all that apply)**

|  | Catching up/ communicating with friends and colleagues |
| --- | --- |
|  | Networking |
|  | Socializing |
|  | Sharing knowledge |
|  | News update |
|  | Entertainment |
|  | Clinical updates |
|  | Research updates |
|  | Event updates |
|  | Politics |
|  | Job updates |
|  | Internet-based shopping |
|  | Others; please specify _________________________________ |

1. **Do you use social media for work?**

- Yes (proceed to Q16)
- No

**Why do you not use social media in your professional life? (select all that apply)**

- Lack of knowledge on how to use social media
- Not suitable for my needs
- Concerned about privacy
- Concerned regarding its safety
- Concerned regarding the validity of the information on social media
- Concerned about negative impact on my reputation
- Not interested
- No time
- Others; please specify ___________________________________

(Proceed to Q18)

**Which social media platform do you use for work and how frequently do you use it? (select all that apply)**

**Please state the number of hours using the social media platform. This includes browsing, not just posting.**

|  | **Social Media Platform** | **Average hour per weekly use** | **Never** |
| --- | --- | --- | --- |
|  | Facebook |  |  |
|  | YouTube |  |  |
|  | Whatsapp |  |  |
|  | Messenger |  |  |
|  | Instagram |  |  |
|  | QQ |  |  |
|  | Tumblr |  |  |
|  | Qzone |  |  |
|  | Tik Tok |  |  |
|  | Sina Weibo |  |  |
|  | Twitter |  |  |
|  | Reddit |  |  |
|  | Baidu Tieba |  |  |
|  | LinkedIn |  |  |
|  | Viber |  |  |
|  | Snapchat |  |  |
|  | Pinterest |  |  |
|  | Line |  |  |
|  | Telegram |  |  |
|  | Medium |  |  |
|  | Others; please specify __________________ |  |  |

1. **What are the main reasons for you to use social media in a work-related manner?**

|  | A source of information |
| --- | --- |
|  | A source of new resources |
|  | Learning new skills |
|  | To expand my professional network |
|  | To establish a professional web-based presence |
|  | To interact with international colleagues |
|  | To pass time |
|  | Others; please specify _________________________________ |

1. **If we are to harness social media as communication tools for rheumatology, which of the following group(s) would be most likely to benefit in your opinion? (select all that apply)**
   - Patients
   - Caregivers
   - Healthcare providers in the community
   - Healthcare providers in acute hospital
   - Researchers
   - Others; please specify _________________________________
2. **If we are to harness social media as communication tools for rheumatology, which age group(s) would best be targeted? (select all that apply)**
   - Below 30 years
   - 30 – 39 years
   - 40 – 49 years
   - 50 – 59 years
   - 60 – 69 years
   - 70 years and above
3. **What are the top 3 areas in which social media would be useful for rheumatology?**

**Please rank your top 3 choices with 1 being the most useful, 2 being the second most useful and 3 being the third most useful.**

- - Education for medical students
  - Education for patients
  - Education for physicians
  - Research
  - International collaboration

Disease activity monitoring

- - Shared decision making
  - Telemedicine

None

Others (please specify _______________________________)

1. **In which area(s) does your employer’s organisation currently use social media? (select all that apply)**
   - Education for medical students
   - Education for patients
   - Education for physicians
   - International collaboration
   - Research

Disease activity monitoring

- - Shared decision making
  - Telemedicine

None

Others (please specify _______________________________)

1. **What would be the main challenges in the wider adoption of social media for rheumatology? (select all that apply)**
   - Time investment
   - Adoption by health care providers
   - Adoption by patients
   - Infrastructure development of technological approaches
   - Supervision and follow-up
   - Cost investment
   - Legal grounds
   - Security concern
   - Confidentiality concern
   - Information anarchy
   - Workplace acceptance and support
   - Inefficiency
   - Lack of skills
   - Others; please specify _______________________________________

**Do you feel that there is potential for social media to be used in any other way aside from how it is currently used? Please explain further.**

- Yes
- No

**Comments:**

**In case you were not using social media before, would you consider using social media in the future?**

- Yes
- No
- Not applicable
