# Supplementary material for: Social Media Use Among Members of the Assessment of Spondyloarthritis International Society: Results of a Web-Based Survey
Source: J Med Internet Res. 2023 Jan 10;25:e39155. doi: 10.2196/39155 (PMC9875001; doi:10.2196/39155)
Supplement: Multimedia Appendix 2 [file jmir_v25i1e39155_app2.docx]

Multimedia Appendix 2: Geographical distribution of all ASAS members ^a^

| **Geographical region** | **ASAS members, n (%)** | **Respondents in this study, n (%)** | **p-value** |
| --- | --- | --- | --- |
| Africa | 3(1.44) | 4 (2.6) | 0.621 |
| Asia and Pacific | 36(17.31) | 22 (14.1) |  |
| Europe | 129(62.02) | 104 (66.7) |  |
| North America | 29(13.94) | 16 (10.3) |  |
| South America | 11(5.29) | 10 (6.4) |  |

ASAS: Assessment of SpondyloArthritis international Society

a. Pearson’s chi-squared test was used to compare the geographical distribution of all ASAS members and respondents in this study
